# Molecular mechanism of tumour necrosis factor alpha regulates hypocretin (orexin) expression, sleep and behaviour

**DOI:** 10.1111/jcmm.14566

**Published:** 2019-08-06

**Authors:** Shuqin Zhan, Pulin Che, Xue‐ke Zhao, Ning Li, Yan Ding, Jianghong Liu, Spring Li, Karyn Ding, Lynn Han, Zhaoyang Huang, Liyong Wu, Yuping Wang, Meng Hu, Xiaosi Han, Qiang Ding

**Affiliations:** ^1^ Department of Neurology, Xuanwu Hospital Capital Medical University Beijing China; ^2^ Beijing Key Laboratory of Neuromodulation Beijing China; ^3^ Department of Medicine University of Alabama at Birmingham Birmingham AL USA; ^4^ Neurology University of Alabama at Birmingham Birmingham AL USA

**Keywords:** Alzheimer, hypocretin, orexin, Parkinson, rapid eye movement sleep behaviour disorder, TNF

## Abstract

Hypocretin 1 and hypocretin 2 (orexin A and B) regulate sleep, wakefulness and emotion. Tumour necrosis factor alpha (TNF‐α) is an important neuroinflammation mediator. Here, we examined the effects of TNF‐α treatment on hypocretin expression in vivo and behaviour in mice. TNF‐α decreased hypocretin 1 and hypocretin 2 expression in a dose‐dependent manner in cultured hypothalamic neurons. TNF‐α decreased mRNA stability of prepro‐hypocretin, the single precursor of hypocretin 1 and hypocretin 2. Mice challenged with TNF‐α demonstrated decreased expression of prepro‐hypocretin, hypocretin 1 and hypocretin 2 in hypothalamus. In response to TNF‐α, prepro‐hypocretin mRNA decay was increased in hypothalamus. TNF‐α neutralizing antibody restored the expression of prepro‐hypocretin, hypocretin 1 and hypocretin 2 in vivo in TNF‐α challenged mice, supporting hypocretin system can be impaired by increased TNF‐α through decreasing hypocretin expression. Repeated TNF‐α challenge induced muscle activity during rapid eye movement sleep and sleep fragmentation, but decreased learning, cognition and memory in mice. TNF‐α neutralizing antibody blocked the effects of TNF‐α; in contrast, hypocretin receptor antagonist enhanced the effects of TNF‐α. The data support that TNF‐α is involved in the regulation of hypocretin expression, sleep and cognition. The findings shed some lights on the role of neuroinflammation in neurodegenerative diseases including Alzheimer's disease and Parkinson's disease.

## 1 INTRODUCTION

Hypocretin 1 and hypocretin 2 (aka orexin A and B, OXA and OXB) are important neuropeptides that were first described in 1998 almost simultaneously by two independent research groups.^1^, ^2^ Prepro‐hypocretin is the single precursor of hypocretin 1 and hypocretin 2, which generates hypocretin 1 and hypocretin 2 through proteolytic cleavage.^1^, ^2^ Prepro‐hypocretin is expressed mainly in hypothalamic neurons and in testis.^1^, ^2^, ^3^ So far, two hypocretin receptors are identified, hypocretin receptor 1 (HcrtR1) and hypocretin receptor 2 (HcrtR2) (aka, orexin receptor 1/OX1R, and orexin receptor 2/OX2R, respectively); they are G‐protein‐coupled receptors.^2^, ^4^, ^5^ Both receptors share over 60% homology in their amino acid sequences.^4^ Both hypocretin 1 (OXA) and hypocretin 2 (OXB) binds to HcrtR2 (OX2R), whereas hypocretin 1 (OXA) preferentially binds HcrtR1 (OX1R).^2^, ^6^ Hypocretin system imbalance or disorders have been blamed for sleep disorder associated with multiple diseases, narcolepsy, emotional disorders, such as depression.^4^, ^7^, ^8^, ^9^ Neurodegenerative diseases often associate with sleep disorders.^10^, ^11^, ^12^ It is less obvious about the direct role of hypocretins (orexins) in neurodegenerative diseases including Parkinson's disease (PD) and Alzheimer's disease (AD), as well as in patients with rapid eye movement (REM) sleep behaviour disorder (RBD).

Tumour necrosis factor alpha (TNF‐α) is a potent pro‐inflammation glycoprotein cytokine which was identified in 1975.^13^ It is well documented that TNF‐α has a critical role in host defence and wound healing; however, when dysregulated, TNF‐α can contribute to the pathogenesis of various diseases, particularly persistent inflammatory responses causing cell stress and tissue damage.^14^, ^15^, ^16^ The activation of TNF‐α signalling cascade has been associated with essential responses for homeostasis and also convincingly associated with the progression of certain diseases that mainly affect brain functions. Published data have linked the activation of TNF‐α signalling to multiple brain disorders, such as the development of narcolepsy, depression, neurodegenerative diseases such as AD and PD, and multiple sclerosis.^17^, ^18^, ^19^, ^20^ Published data have implicated a role of TNF‐α in sleep and wakefulness cycle, as well as fatigue, during infections.^21^ Our previous study has demonstrated that TNF‐α regulates hypocretin system in vitro in cultured cells.^22^ The role of TNF‐α in the regulation of hypocretin system in vivo and the molecular mechanism involved are unknown to the field.

In the present study, we investigated the effect of TNF‐α on expression of hypocretin 1 and hypocretin 2 in vitro and in vivo. The effects of TNF‐α on hypocretin expression in vivo, muscle activity during REM sleep (RBD), learning, cognition and memory were examined in mice. The findings support an important role of TNF‐α in the regulation of hypocretin system, sleep, and cognition and learning. The findings also shed some lights on the role of neuroinflammation on some aspects of neurodegenerative diseases including Alzheimer's disease and Parkinson's disease.

## 2 MATERIALS AND METHODS

### 2.1 Reagents

The following purified antibodies were purchased: hypocretin 1 (Santa Cruz Biotechnology sc‐80263, 1:250), hypocretin 2 (Abcam, ab229714, 1:500), beta‐actin (β‐actin) (Santa Cruz Biotechnology, SC‐8432, 1:1000), prepro‐hypocretin (Millipore and Sigma, AB3096, 1:500), P38 (Cell Signaling, #9212, 1:1000) and phospho‐p38 (Cell Signaling, #4511, 1:1000). Recombinant tumour necrosis factor alpha (TNF‐α) was obtained from R&D Systems. TNF‐α neutralizing antibody was purchased from BD Biosciences and Cell Signaling (11 969, used as indicated). Rat IgG was purchased from Jackson ImmunoResearch (415 005 166, used as indicated). The hypocretin (orexin) receptor 1 (HcrtR1/OX1R) antagonist SB334867 was purchased from Tocris (United Kingdom). All other reagents were purchased from Cell Signaling, Thermo Fisher Scientific (P‐SUWANEE, GA), Sigma‐Aldrich or Bio‐Rad.

### 2.2 Western blotting

Protein production was determined by Western blot analysis as described by us previously.^23^ Cells or tissues were lysed in 1% NP‐40 lysis buffer containing the following inhibitors, 100 μmol/L PMSF, 10 μg/mL aprotinin, 10 μg/mL leupeptin, 100 μmol/L sodium vanadate and 20 μg/mL TLCK. Protein concentration of whole tissue or cell lysate was determined by BCA kit (Pierce). Equivalent micrograms of lysates were electrophoresed on SDS‐PAGE, transferred to Immobilon‐P membrane (Millipore Corp.), probed with indicated antibodies and developed with ECL system (Pharmacia Biotech). The beta‐actin protein level was used as a loading control. Quantitative analysis (densitometry) of Western blots was performed by calculating the relative density (pixel density) of the immunoreactive bands after acquisition of the blot image (scanning) and analysis with Adobe Photoshop software as described.^24^ The background of densitometric reading on the ECL‐developed film was subtracted.

### 2.3 Real‐time quantitative PCR

Expression of prepro‐hypocretin mRNA was determined by real‐time quantitative RT‐PCR as described by us previously.^25^ Total RNA was extracted from cells using the RNeasy Mini Kit (Qiagen, Inc), and 1 µg of total RNA was reverse‐transcribed to cDNA for real‐time quantitative RT‐PCR as described previously.^26^ Quantitative RT‐PCR analysis was carried out with the SYBR^®^ Green PCR Master Mix (Applied Biosystems) using the Bio‐Rad iQ5 Real‐Time PCR Detection System, according to the manufacturer's instructions. The quantitative RT‐PCR was performed using 4 µL of the synthesized cDNA, 12.5 µL of SYBR^®^ Green PCR Master Mix, 1 µL of 10 µmol/L primer mix and 7.5 µL of water in a final 25 µL volume. Samples were assayed in triplicate, and the values were normalized to the relative amounts of beta‐actin mRNA level. Primer sequences were as described.^22^ Primers for prepro‐hypocretin: sense 5′‐GCCGTCTCTACGAACTGTTG‐3′ and antisense 5′‐GAGGAGAGGGGAAAGTTAG‐3′; for control focal adhesion kinase (FAK): sense 5′‐CCTTAACAATGCGCCAGTTT‐3′ and antisense 5′‐CCAGATACGCGAGTGCTGTA‐3′; and for beta‐actin: sense 5′‐GTGGGTATGGGTCAGAAGGA‐3′ and antisense 5′‐AGCGCGTAACCCTCATAGAT‐3′. All primers were purchased from Fisher Scientific.

### 2.4 Prepro‐hypocretin mRNA stability analysis

Prepro‐hypocretin mRNA stability and half‐life were determined by culturing primary murine hypothalamic neuron cells in medium with actinomycin D (10 μg/mL) to block transcription as described by us previously.^25^ Briefly, cells were treated with actinomycin D, then followed by TNF‐α or vehicle treatments for the indicated time, and harvested. Total RNA was collected, and the amounts of prepro‐hypocretin mRNA at each time‐point were quantified by real‐time RT‐PCR and normalized to the amounts of β‐actin mRNA at the same time‐point.

### 2.5 Cell culture

Hypothalamic tissue removal and primary hypothalamic neurons isolation and culture were basically described previously^27^, ^28^, ^29^, ^30^ with minor modifications. Briefly, hypothalamic tissue was removed from 11‐ to 13‐week‐old C57BL6 mice. After dissection, tissues were first placed into ice‐cold Dulbecco's modified Eagle's medium (DMEM) supplemented with 1% foetal bovine serum (FBS) and 25 mmol/L Hepes at pH7.4. Hypothalamic tissues then were dispersed into single cells by controlled enzyme treatment as previously described.^27^, ^29^ Hypothalamic tissues were digested with papain (Worthington), and hypothalamic neurons were purified with gradient steps,^27^ plated onto chambered glass slides coated with laminin and poly‐D‐lysine (Sigma) and then cells were cultured in neurobasal cell culture medium supplemented with B27 supplement (GIBCO, 4%), 2 mmol/L l‐glutamine, 4.5 mg/mL glucose, 100 µg/mL gentamicin and 100 units/mL penicillin/streptomycin/gentamicin at 37 degree as described.^24^

### 2.6 TNF‐α treatment and behavioural phenotypes (sleep, muscle activity, learning, cognition and memory)

The research has been approved by local Animal Care and Use Committee (IACUC #09243 and #21589). For the effect of TNF‐α on hypocretin expression in hypothalamus, one‐time intraperitoneal injections of TNF‐α at indicated dose or control vehicle saline were performed. TNF‐α levels in serum were examined 1 hour after injection. TNF‐α levels in hypothalamic tissues were examined at 6 hour after injection. For neutralizing studies, mice received intravenous injection of rat TNF‐α neutralizing antibody (1 mg/kg whole body weight) or control rat IgG 30 minutes before TNF‐α injection. For the effect of blocking hypocretin signalling, the HcrtR1/OX1R antagonist SB334867 was used. SB334867 was dissolved in dimethyl sulphoxide (DMSO) and diluted in sterile saline (30 mg/kg) for intraperitoneal administration 30 minutes before TNF‐α injection. For the effects of TNF‐α on sleep, muscle activity, learning, cognition, memory, intraperitoneal injection of TNF‐α or control saline were performed at 5 pm, 3 times per week for three weeks. For the effect of TNF‐α on sleep, mice (12‐ to 13‐week‐old C57BL6, approximately 21 g, male and female, Jackson Laboratory., stock # 000664) were studied by video tracking similarly as described^31^ and the motion was tracked by a computerized video tracking system (EthoVision, Noldus), or anaesthetized with ketamine and xylazine and implanted with three electroencephalographic (EEG) electrodes in the skull over the parietal cortex and three electromyogram (EMG) electrodes in the muscle of the dorsal neck, respectively, as described previously.^32^, ^33^ The electrodes with attached wires were fixed to the skull with dental cement, and mice were allowed for recovery for 2 weeks with a 12‐hour light/dark cycle (lights on at 5 am and off at 5 pm). All animals were identified by earmarks and numbered accordingly. Using a table of random numbers, animals were randomly divided into experimental groups. Animals from experimental groups were always treated and assessed first, followed by control groups. During experiments and analysis, the investigators were blinded to genotype and experimental group. Animals were identified by earmarks and got numbers, which were announced to the investigator only after finishing experiments and analysis. Data were recorded and analysed by sleep sign system according to the criteria described previously.^32^

For the effect of TNF‐α on learning, cognition, and memory, spatial reference learning and memory abilities were assessed in a modified open field Morris water maze basically as described previously.^34^, ^35^, ^36^ The Morris water maze is one of the widely used tasks in behavioural neuroscience for studying the neural mechanisms of spatial learning, cognition and memory.^34^ Briefly, the water maze is a blue circular 1.2 m pool filled with clear water. The room contained abundant extra‐maze visual cues for orientation. The mice are gently placed in the water facing to and next to the wall of the pool and allowed to explore freely in order to locate a fixed escape platform, hidden 0.5 cm beneath water level in the middle of the NE quadrant. The mouse's task throughout the experiment is to find and to escape onto the platform. In each trial, mice were placed in water from one of different positions (north, east, south and west) in a pseudorandom order. Then, each mouse was allowed to swim until it found the hidden platform or until 60 seconds had elapsed, at which point the mouse was guided to the platform, where it remained for 10 seconds before being returned to a cage containing paper towels to dry. The inter‐trial interval was 120 seconds. In the probe trial, the platform was removed from the pool, and animals were allowed to swim for 60 seconds. On day 6, a probe trial was conducted by removing the platform and placing the mouse next to and facing the North side. The time spent in the target quadrant was measured in a single 60‐second trial. The escape latency and swim path length were recorded for four trials daily for 5 days by a computerized video tracking system (EthoVision, Noldus).

Novel object recognition (NOR) model is widely used as a simple behavioural assay for the investigation of alterations in recognition memory. To examine the effect of TNF‐α on NOR, the analysis was performed basically as described previously.^37^, ^38^ Briefly, the task procedure consists of three phases: habituation, familiarization and test phase. The experimental context remains the same during all phases. Between each trial, the walls, floors and objects were wiped with ethanol to eliminate odour residues. On day 1, mice were placed in the empty arena for 10 minutes free exploring to allow habituation to the environment. On day 2, during the familiarization phase, a mouse was placed in the same arena containing two identical objects and allowed free exploring for 10 minutes. Twenty hours later, during the test phase, the animal was returned to the same arena for a duration of 10 minutes, where one of the identical objects was replaced by a novel object. The time each animal spent on exploring familiar and novel objects was documented and scored using fully automated EthoVision video tracking software. The percentage of time each animal spent on exploring the novel object over the total duration on exploring both objects was determined as the recognition index.

### 2.7 Retrospective analysis RBD frequency in Parkinson's disease (PD), Alzheimer's disease (AD) and narcolepsy patients

The research has been approved by local Institutional Review Board (IRB #N13106548), and informed consent was obtained. The retrospective analysis of RBD frequency was based on using unidentified records of questionnaire, which is a widely used and well‐established single question questionnaire with a sensitivity of 94% and a specificity of 87% for RBD.^39^, ^40^, ^41^ The screening questionnaire by using the following question for the bed partner: ‘Have you ever seen the patient appear to “act out his/her dreams” while sleeping (punched or flailed arms in the air, shouted or screamed)?’ or to the patient: ‘Have you ever been told or seen that you seem to “act out your dreams” while sleep, for example, punching, flailing your arms in the air, shouted or screamed?’ The ‘yes’ subjects, if agreed voluntarily, were recruited for further RBD sleep analysis, and the findings were included in the unidentified records. A small cohort of clinical identified Parkinson's disease (PD, 52 subjects), Alzheimer's disease (AD, 26 subjects) and narcolepsy (28 subjects) were screened by the questionnaire and generated the unidentified record for further analysis.

### 2.8 Statistical analysis

Data were analysed using the unpaired or paired *t* test analysis (for comparisons between two groups) (Sigma Plot, SPSS Inc). Data points outside the 95% confidence interval were treated as outliers and excluded from the data analysis. A *P* value of <0.05 was considered statistically significant.

## 3 RESULTS

### 3.1 TNF‐α treatment decreases hypocretin 1 and hypocretin 2 protein production in a dose‐dependent manner in primary hypothalamic neurons

**Figure 1:**
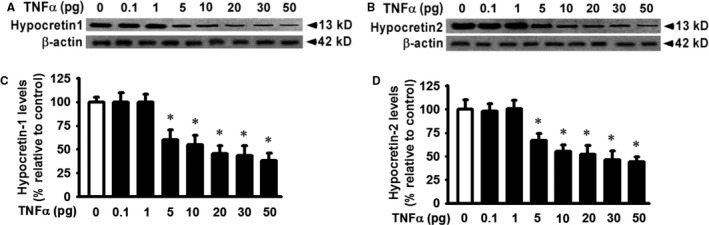
TNF‐α decreases hypocretin 1 and hypocretin 2 protein production in a dose‐dependent manner. Primary hypothalamic neuron cells were cultured in the absence (control vehicle) or presence of TNF‐α at indicated dose (0‐50 pg/mL final concentration) for 24 h at 37°C. Cells were lysed, and equivalent amount of whole cell detergent lysates was Western blotted with the indicated antibodies for (A) hypocretin 1 and (B) for hypocretin 2. (C) Densitometry of hypocretin 1 protein production as shown in Panel A (normalized to actin and relative to the controls), (D) densitometry of hypocretin 2 protein production as shown in Panel B. n = 4. ^*^Represents *P* < 0.01 for TNF‐α‐treated cells compared to vehicle‐treated control

We have previously reported that TNF‐α decreased hypocretin expression in rat B35 neuroblastoma cells in which exogenous hypocretin was overexpressed mediated by retroviral vectors.^22^ To investigate the role of TNF‐α in the regulation of hypocretin production in primary hypothalamic neurons, where is the main source of endogenous hypocretin, primary hypothalamic neurons were serum starved for 12 hours and then followed by TNF‐α treatments at the indicated concentration (Figure 1). TNF‐α treatment induced a decrease of hypocretin 1 protein level in primary hypothalamic neurons in a dose‐dependent manner when compared to that in controls (Figure 1A and C, significant decrease starting at 5 pg/mL, *P* < 0.01). TNF‐α treatment simultaneously decreased hypocretin 2 protein levels in a dose‐dependent manner when compared to that in controls (Figure 1B and D, significant decrease starting at 5 pg/mL, *P* < 0.01).

### 3.2 TNF‐α treatment inhibits the expression of prepro‐hypocretin, the single precursor of hypocretin 1 and hypocretin 2, through destabilizing prepro‐hypocretin mRNA in primary hypothalamic neurons

**Figure 2:**
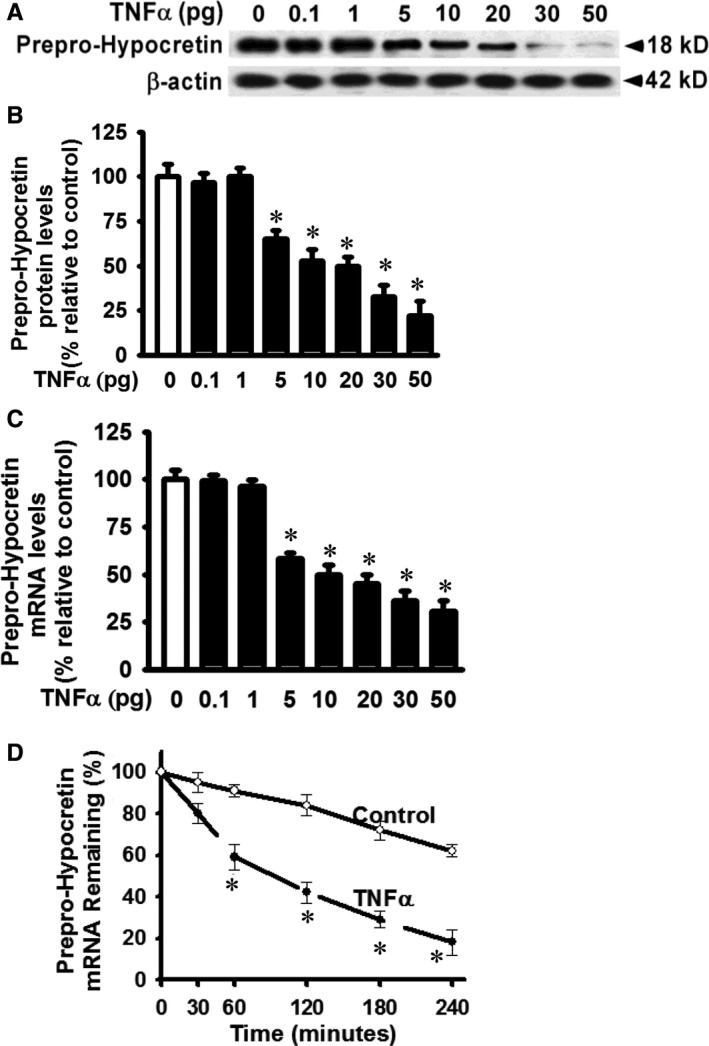
TNF‐α inhibits prepro‐hypocretin protein production and destabilizes prepro‐hypocretin mRNA in primary hypothalamic neurons. Neuron cells were cultured in the absence (control vehicle) or presence of TNF‐α at indicated dose (0‐50 pg/mL final concentration) and lysed, and equivalent amount of whole cell detergent lysates was Western blotted with the indicated antibodies for (A) prepro‐hypocretin, (B) densitometry of prepro‐hypocretin protein production in Panel A, (C) cells were treated as in Panel A and total RNA was extracted. Prepro‐hypocretin mRNA levels were analysed by quantitative real‐time RT‐PCR (normalized to beta‐actin and relative to that in vehicle‐treated cells). (D) Primary hypothalamic neurons were treated with actinomycin D (10 μg/mL) to inhibit new transcription and then treated with control vehicle (control) or with TNF‐α (30 pg/mL final concentration) for the indicated time range (minutes). Total RNA was extracted to determine the amount of prepro‐hypocretin mRNA at each indicated time‐point (for stability and half‐life) by real‐time quantitative RT‐PCR. Bottom line denotes the results from TNF‐α‐treated cells. n = 4. ^*^Represents *P* < 0.01 for TNF‐α‐treated cells compared to vehicle‐treated control

Prepro‐hypocretin is the single precursor of both hypocretin 1 and hypocretin 2; prepro‐hypocretin generates hypocretin 1 and hypocretin 2 through proteolytic cleavage.^1^, ^2^ Protein production of prepro‐hypocretin was inhibited in primary cultured hypothalamic neurons in response to TNF‐α treatment (Figure 2A). The effects of TNF‐α on reduction of prepro‐hypocretin protein levels were in a dose‐dependent manner (Figure 2A and 2B), similar to TNF‐α‐induced reduction of hypocretin 1 and hypocretin 2 protein levels in Figure 1. In response to TNF‐α treatment, prepro‐hypocretin mRNA levels were decreased in a dose‐dependent manner (Figure 2C). To understand the mechanism resulting in reduction of both mRNA and protein levels of prepro‐hypocretin in response to TNF‐α, we examined the decay and degradation of prepro‐hypocretin mRNA. Prepro‐hypocretin mRNA was significantly destabilized starting at about 60 minutes after TNF‐α treatment (30 pg/mL) (Figure 2D). The decreased hypocretin 1 and hypocretin 2 protein production in response to TNF‐α was due to destabilized prepro‐hypocretin mRNA, the common precursor of both hypocretin 1 and hypocretin 2.

### 3.3 Blocking TNF‐α signalling increases prepro‐hypocretin protein level and enhances prepro‐hypocretin mRNA stability in primary hypothalamic neurons

**Figure 3:**
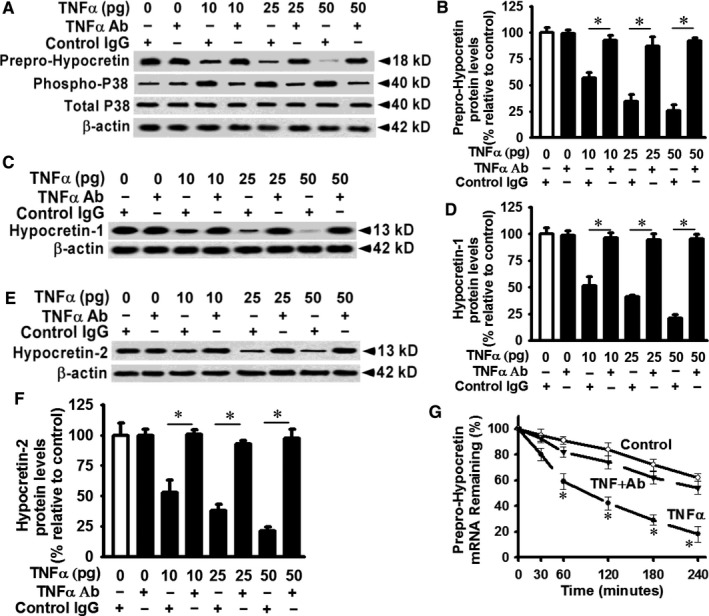
TNF‐α neutralizing antibody blocks TNF‐α signalling, increases prepro‐hypocretin protein production and inhibits prepro‐hypocretin mRNA decay. Primary hypothalamic neurons were pre‐treated with TNF‐α blocking antibody (1 µg/mL) 20 min before TNF‐α (0‐50 pg/mL) or vehicle treatments as in Figure 1. Cells were lysed, and equivalent amount of whole cell detergent lysates was analysed by Western blot for (A) prepro‐hypocretin and TNF‐α‐induced p38 phosphorylation, (B) densitometry of prepro‐hypocretin protein production in panel A, (C) hypocretin 1 protein production, (D) densitometry of hypocretin 1 protein production in panel C, (E) hypocretin 2 protein production, (F) densitometry of hypocretin 2 protein production in panel E, (G) Prepro‐hypcretin mRNA half‐life and expression were examined as described in Figure 2 at each indicated time‐point. The middle line denotes the results for the effect of neutralizing antibody in TNF‐α treated (30 pg/mL final concentration) cells. n = 4. ^*^Represents *P* < 0.01 for TNF‐α‐treated cells compared to vehicle‐treated control

Pre‐treatment of TNF‐α neutralizing antibody blocked TNF‐α‐induced reduction of prepro‐hypocretin protein levels in primary hypothalamic neurons (Figure 3A and B). P38 mitogen‐activated protein kinase (MAPK) is a known downstream signalling protein of TNF‐α.^42^ TNF‐α antibody inhibited TNF‐α‐induced p38 MAPK activation (Figure 3A), supporting the effect of neutralizing antibody is specific towards TNF‐α signalling. Pre‐treatment of TNF‐α neutralizing antibody restored the protein levels of hypocretin 1 protein levels (Figure 3C and D) and hypocretin 2 protein levels (Figure 3E and F). Control IgG treatments had no effects on TNF‐α‐induced p38 MAPK activation and TNF‐α‐induced reduction of hypocretin protein production (Figure 3A and B). As shown in Figure 2, TNF‐α treatment destabilizes prepro‐hypocretin mRNA. The TNF‐α‐induced decay of prepro‐hypocretin mRNA was blocked by TNF‐α neutralizing antibody in primary hypothalamic neurons (Figure 3G). The half‐life of prepro‐hypocretin mRNA was about 90 minutes in TNF‐α‐treated cells (Figure 3G). In response to TNF‐α neutralizing antibody, the half‐life of prepro‐hypocretin mRNA was significantly extended (above 240 minutes), comparable to that in controls (middle line in Figure 3G).

### 3.4 TNF‐α significantly decreases the mRNA and protein levels of prepro‐hypocretin in vivo in hypothalamus in mice

**Figure 4:**
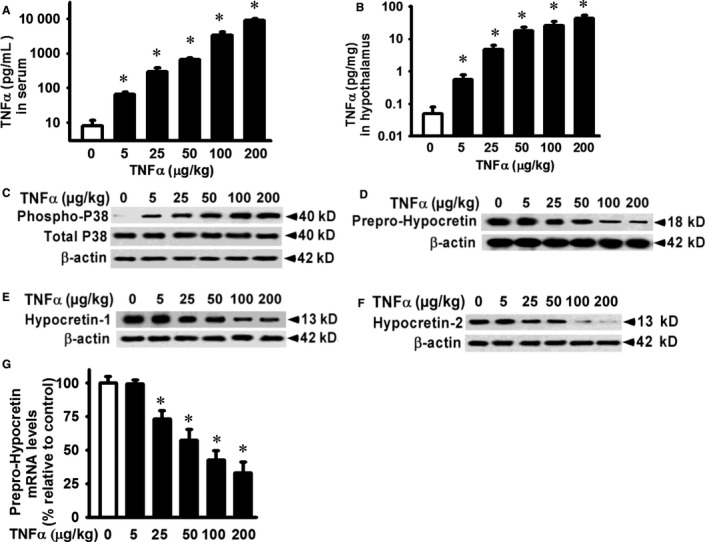
TNF‐α decreases prepro‐hypocretin protein production and destabilizes prepro‐hypocretin mRNA in hypothalamus. Mice were intraperitoneally injected with TNF‐α at indicated amount (5‐200 µg/kg whole body weight) or control vehicle in 60 µL saline solution. TNF‐α levels in (A) serum and (B) hypothalamic tissue lysates were examined by ELISA kits. Hypothalamic tissues were detergent lysed, and equivalent amount of whole tissue lysates was analysed by Western blot for (C) p38 MAPK phosphorylation, (D) prepro‐hypocretin protein production, (E) hypocretin 1 protein production, (F) hypocretin 2 protein production and (G) total RNA was extracted from hypothalamic tissues. Prepro‐hypocretin mRNA expression was analysed by quantitative real‐time RT‐PCR (normalized to beta‐actin and relative to that in mice injected with control vehicle). Data are presented as mean ± SE (n = 6). ^*^Represents *P* < 0.01 for TNF‐α‐treated mice compared to vehicle‐treated control

To examine the effects of TNF‐α on prepro‐hypocretin expression in vivo in hypothalamus, TNF‐α or control saline solution was intraperitoneally injected (Figure 4). Serum TNF‐α levels were increased in a dose‐dependent manner after injection (Figure 4A). TNF‐α levels in hypothalamic tissues after injection were also increased in a dose‐dependent manner (Figure 4B), similar to the increased pattern in serum but in a much less scale. To understand the biological effects of TNF‐α on hypocretin system, we first examined the activation of p38 MAPK in hypothalamic tissues. P38 MAPK is not only a known downstream signalling protein of TNF‐α, but also plays an important role in inflammation.^42^ TNF‐α induced p38 phosphorylation in hypothalamic tissues at the lowest tested dose (5 µg/kg, Figure 4C). Prepro‐hypocretin protein production in hypothalamic tissues was decreased in response to TNF‐α, starting at the dose of 25 µg/kg (Figure 4D). Consequently, the protein production of hypocretin 1 and hypocretin 2 was decreased in hypothalamic tissues in response to TNF‐α treatment (Figure 4E and F), likely due to the decreased protein production of precursor protein, prepro‐hypocretin. Prepro‐hypocretin mRNA expression was significantly decreased in hypothalamic tissues in response to TNF‐α treatment, starting at 25 µg/kg TNF‐α dose and then progressively decreased in an inverse relationship with the amount of TNF‐α treated (Figure 4G).

### 3.5 TNF‐α blocking antibody restored prepro‐hypocretin protein production and mRNA stability in hypothalamus in mice

**Figure 5:**
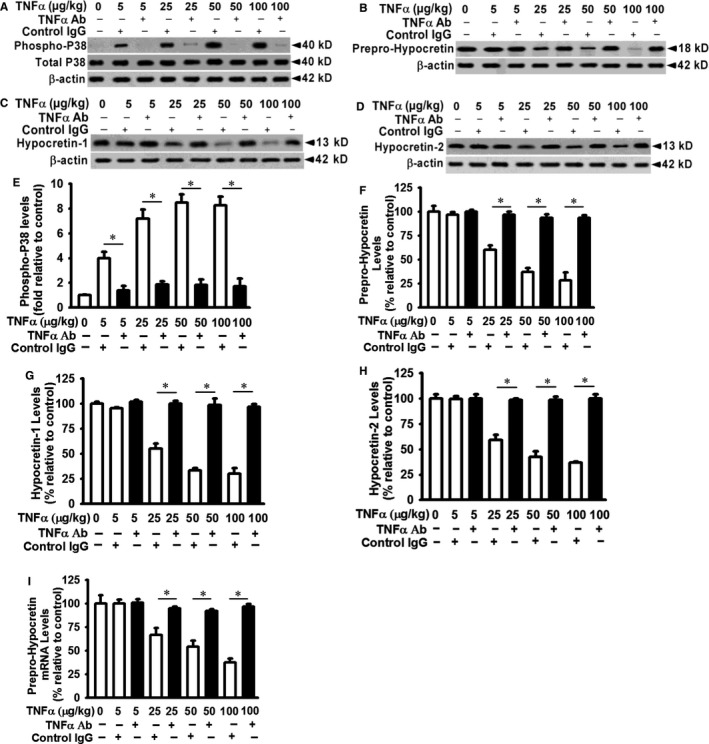
TNF‐α neutralizing antibody restores prepro‐hypocretin protein production and increases prepro‐hypocretin mRNA stability in TNF‐α‐treated mice. Mice received intravenous injection of TNF‐α neutralizing antibody (1 mg/kg whole body weight) or control IgG antibody (1 mg/kg whole body weight) 30 min before TNF‐α (5‐100 µg/kg whole body weight) or control vehicle injection as described in Figure 4. Hypothalamic tissues were detergent lysed, and equivalent amount of whole tissue lysates was analysed by Western blot for (A) p38 MAPK phosphorylation, (B) prepro‐hypocretin protein production, (C) hypocretin 1 protein production, (D) hypocretin 2 protein production, (E‐H) densitometry analysis for Panels A‐D, respectively and (I) prepro‐hypocretin mRNA expression in hypothalamic tissues was analysed as in Figure 4. Data are presented as mean ± SE (n = 5). ^*^Represents *P* < 0.01 for TNF‐α‐treated mice compared to vehicle‐treated control

TNF‐α significantly decreases the expression of hypocretin 1 and hypocretin 2 in hypothalamus in mice (Figure 4). To examine the effect of blocking TNF‐α signalling on hypocretin expression, mice received intravenous injection of TNF‐α neutralizing antibody (1 mg/kg whole body weight) or control IgG antibody (1 mg/kg whole body weight) 30 minutes before TNF‐α injection. Compared to mice receiving control IgG, mice received TNF‐α neutralizing antibody showed decreased p38 MAPK activation in hypothalamic tissue in response to TNF‐α challenge (Figure 5A and E). Prepro‐hypocretin protein production was restored in hypothalamic tissues in mice received TNF‐α neutralizing antibody when compared to that in mice received control IgG in response to TNF‐α challenge (Figure 5B and F). TNF‐α neutralizing antibody treatment blocked TNF‐α‐induced reduction of hypocretin 1 (Figure 5C and G) and hypocretin 2 (Figure 5D and H) in hypothalamic tissues when compared to control IgG‐treated mice. In addition, blocking TNF‐α signalling by neutralizing antibody significantly increased prepro‐hypocretin mRNA expression in hypothalamic tissues in mice (Figure 5I). Increased hypocretin 1 and hypocretin 2 protein production is likely due to increased expression of prepro‐hypocretin mRNA, the common precursor of both hypocretin 1 and hypocretin 2.

### 3.6 Repeated TNF‐α challenge induces sleep fragmentation and muscle activity during rapid eye movement sleep in mice

**Figure 6:**
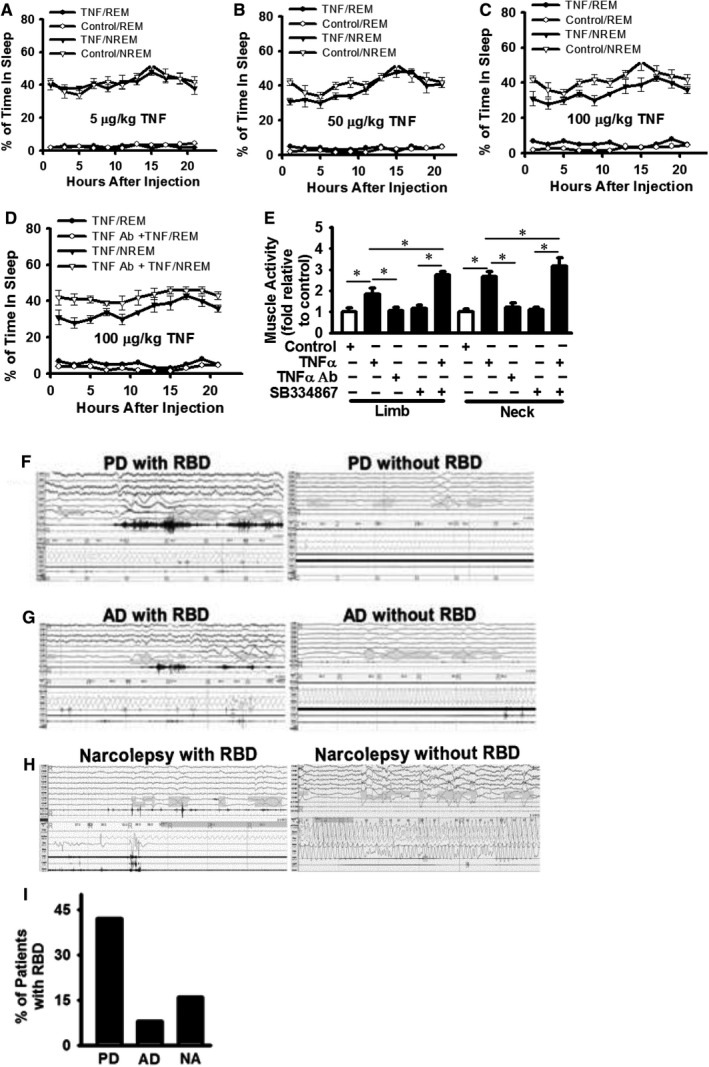
Repeated TNF‐α treatment induces sleep fragmentation and muscle activity during REM sleep in mice. (A‐C) Mice were treated with TNF‐α (5 µg/kg whole body weight in Panel A, 50 µg/kg in Panel B and 100 µg/kg in Panel (C) or control vehicle for 3 weeks (3 time per week) and immediately followed by analysis of the percentage of time in NREM or REM sleep. n = 10. (D) Mice received intravenous injection of TNF‐α neutralizing antibody (1 mg/kg whole body weight) 30 min before TNF‐α (100 µg/kg whole body weight) injection as described in Figure 4. n = 8. (E) Mice treated with high TNF‐α (100 µg/kg) had increased muscle activity during REM sleep when compared to that in mice treated with control vehicle and lower TNF‐α. TNF‐α neutralizing antibody blocked the effect of TNF‐α; in contrast, intraperitoneal injection of SB334867 (30 mg/kg) enhanced the effect of TNF‐α. Data are presented as arbitrate fold to controls. Data are presented as mean ± SD (n = 8 to 9). ^*^Represents *P* < 0.01. (F‐I) Patients with or without RBD (muscle activity during REM sleep). Parkinson's disease (PD) in Panel F, Alzheimer's disease (AD) in Panel G and narcolepsy (NA) patient in Panel H (I) The frequency of PD, AD and NA patients with confirmed RBD by retrospectively analysis of a small cohort of unidentified clinical records

Hypocretin (aka orexin)‐deficient mice show sleep fragmentation with more transitions between wake, non‐rapid eye movement (NREM) and rapid eye movement (REM) sleeps.^43^, ^44^, ^45^ To understand the effects of decreased hypocretin expression induced by TNF‐α treatment, mice were repeatedly challenged with TNF‐α for 3 weeks (3 times per week) and immediately followed by sleep phenotype analysis. Low dose of TNF‐α treatment (5 µg/kg body weight) did not have significant effect on sleep pattern (Figure 6A). In contrast, higher doses of TNF‐α treatment (50 and 100 µg/kg body weight) induced abnormal sleep pattern and sleep fragmentation in mice (Figure 6B and C, respectively). Blocking TNF‐α signalling by TNF‐α neutralizing antibody reversed the effects of TNF‐α on sleep pattern in mice; mice treated with TNF‐α neutralizing antibody showed sleep pattern similar to controls (Figure 6D).

It has been reported that microinjection of hypocretin in the locus coeruleus and pontine inhibitory area results in muscle tone changes.^46^ As the above data demonstrate that TNF‐α decreases hypocretin l and hypocretin 2 levels in brain, it is likely that muscle tone could also be modulated by TNF‐α at least through hypocretin system. To test this, mice were challenged with TNF‐α injection for 3 weeks (3 times per week), and then the consequence on muscle tone was examined (Figure 6E). Muscle activity was induced during REM sleep in mice challenged with higher dose of TNF‐α (100 µg/kg body weight) (Figure 6E); there was no significant difference in muscle activity between other TNF‐α doses and controls (data not shown). TNF‐α neutralizing antibody treatment abrogated the effects of TNF‐α on muscle activity; in contrast, the HcrtR1/OX1R antagonist SB334867 enhanced the effects of TNF‐α on muscle activity (Figure 6E). SB334867 is a functional antagonist and more specific for HcrtR1/OX1R. We speculate that the reduction of OXA in TNF‐α‐treated animals may be more physiologically relevant, in the context of our tested conditions. In human, REM sleep with muscle activity (without atonia) is a key aspect of REM sleep behaviour disorder (RBD) in RBD patients. TNF‐α is a key mediator of neuroinflammation, and neuroinflammation has been associated with neurodegenerative diseases, including Parkinson's disease (PD) and Alzheimer's disease (AD).^47^, ^48^, ^49^ The onset of RBD is suggested to precede the development of neurodegeneration by several years.^50^ The frequency of RBD in these patients has not been well defined. By retrospective analysis of a small cohort of patients, our data suggest that RBD exists in PD, AD and narcolepsy patients, but the percentage of affected patients varies; there were approximately 42% of PD, 8% of AD and 16% of narcolepsy patients with confirmed RBD (Figure 6F‐I).

### 3.7 Repeated TNF‐α challenge decreased learning, cognition and memory in mice

**Figure 7:**
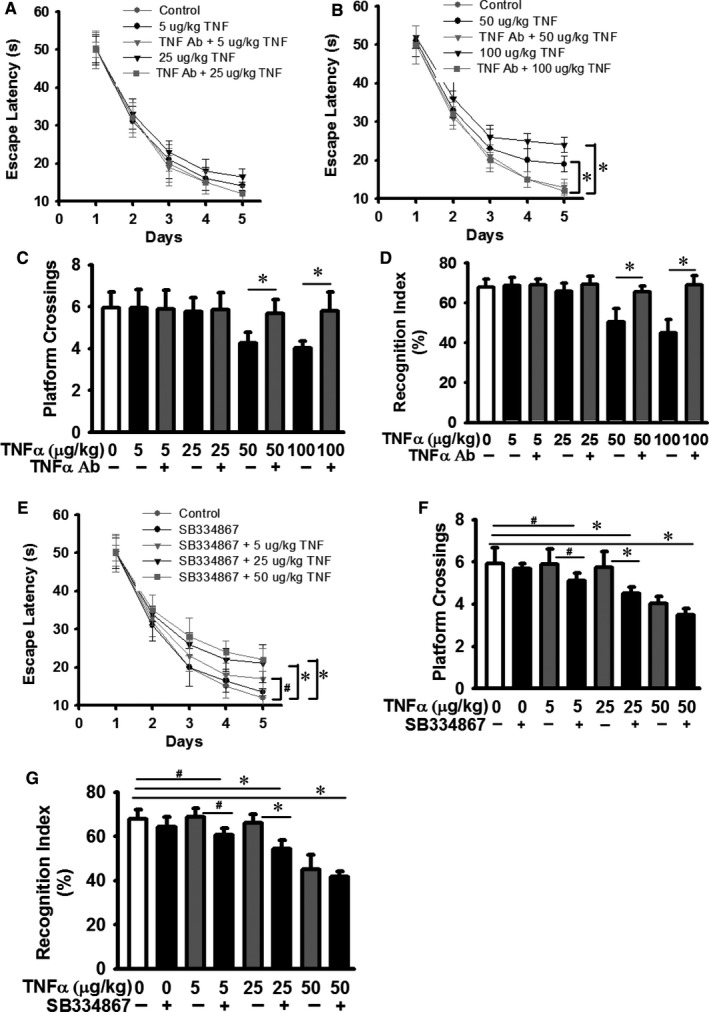
Repeated TNF‐α treatment impairs learning, cognition and memory in mice. Mice received intravenous injection of TNF‐α neutralizing antibody (1 mg/kg whole body weight) or control vehicle 30 min before mice treated with indicated amount of TNF‐α or control vehicle for 3 weeks (3 times per week). (A‐B) Morris water maze assays, (C) Platform crossing frequency assays after removing the platform, (D) Novel object recognition assays as described in Materials and Methods. Mice were intraperitoneally administrated with SB334867 (30 mg/kg, HcrtR1/OX1R antagonist) and followed by TNF‐α treatments, and subjected to (E‐F) Morris water maze and (G) novel object recognition assays. Data are presented as mean ± SD (n = 8). # represents *P* < 0.05 and ^*^Represents *P* < 0.01

Evidence is accumulated to support the involvement of neuroinflammation in neurodegenerative diseases, including Alzheimer's and Parkinson's diseases.^51^, ^52^, ^53^ TNF‐α is a potent pro‐inflammation cytokine^13^ and plays an important role in neuroinflammation.^47^ To determine the effect of repeated TNF‐α challenge on learning, cognition, and memory, the spatial learning and memory ability were examined in mice challenged with TNF‐α or control vehicle by using the Morris water maze and the novel object recognition (NOR) models. In the 5‐day learning test of Morris water maze, mice must learn to use cues to escape onto a platform hidden in a constant location as described in Materials and Methods. Mice treated with higher dose of TNF‐α (50 and 100 µg/kg body weight) required more time to learn to escape than mice treated with control saline vehicle and lower doses of TNF‐α (Figure 7A and B). Mice were also tested in a probe trial during which the platform was removed, and the search patterns were monitored and the time spent in the target quadrant was recorded. Mice treated with higher dose of TNF‐α (50 and 100 µg/kg body weight) showed less platform crossing than mice treated with control saline vehicle and lower doses of TNF‐α (Figure 7C). Additional, mice treated with higher dose of TNF‐α (50 and 100 µg/kg body weight) showed less ability for novel object recognition (NOR) than mice treated with control saline vehicle and lower doses of TNF‐α (Figure 7D). TNF‐α neutralizing antibody treatment abrogated the effects of TNF‐α on increased escape latency (Figure 7B) and decreased platform crossing and NOR (Figure 7C and D). In contrast, blocking hypocretin signalling by SB334867 (HcrtR1/OX1R) antagonist enhanced the effects of TNF‐α on increased escape latency (Figure 7E) and decreased platform crossing and NOR (Figure 7F and G). These observations support that repeated TNF‐α challenge reduces learning, memory and cognition in mice, and hypocretin system is involved in these effects of TNF‐α.

## 4 DISCUSSION

Hypocretin 1 and hypocretin 2 (aka orexin A and B) have critical roles in the regulation of sleep, wakefulness, feeding behaviour and emotion.^7^, ^9^, ^45^ The present study has demonstrated that TNF‐α can impair the hypocretin (orexin) system through decreasing mRNA stability of prepro‐hypocretin in cultured hypothalamic neurons in vitro and in hypothalamus in vivo in mice. Decreased prepro‐hypocretin mRNA stability results in decreased prepro‐hypocretin protein level and subsequently decreased levels of hypocretin 1 and hypocretin 2 in cultured hypothalamic neurons in vitro and in hypothalamus in vivo in mice. TNF‐α neutralizing antibody blocks the inhibitory effects of TNF‐α on hypocretin protein production. Mice treated with TNF‐α neutralizing antibody restore the protein production of both hypocretin 1 and hypocretin 2 in vivo. Functionally, repeated TNF‐α challenge induces muscle activity during REM sleep and sleep fragmentation. Phenotypically, mice with repeated TNF‐α challenge show decreased learning, cognition and memory when compared to that in control mice. The data support an important role of TNF‐α in the regulation of hypocretin system and sleep. As neuroinflammation is involved in neurodegenerative diseases,^51^ the data also shed some lights on the role of TNF‐α on some aspects of neurodegenerative diseases, including Parkinson's disease and Alzheimer's disease.

TNF‐α is involved in the regulation of sleep‐wakefulness behaviour and fatigue during infections and depression.^21^ However, the underlying molecular mechanisms have not been fully elucidated. Our data suggest that TNF‐α is involved in the regulation of sleep‐wakefulness and behaviour at least through its regulation of hypocretin expression. Prepro‐hypocretin is the common precursor of both hypocretin 1 and hypocretin 2. The half‐life of prepro‐hypocretin mRNA is decreased in response to TNF‐α (Figure 2); this is likely the involved molecular mechanism that reduces mRNA expression of prepro‐hypocretin, hypocretin 1 and hypocretin 2 in cultured hypothalamic neurons in vitro and in hypothalamus in mice in vivo in response to TNF‐α challenge (Figures 2 and 3). TNF‐α neutralizing antibody blocked the inhibitory effect of TNF‐α on hypocretin expression (Figures 3 and 5), supporting the specific TNF‐α effect on hypocretin system. Others have reported that TNF‐α can negatively regulate gene expression. TNF‐α treatment decreases the expression of both endothelial nitric oxide synthase and argininosuccinate synthase in aortic endothelial cells.^54^ Based on our current data, the molecular mechanism involved in TNF‐α‐induced down‐regulation of hypocretins is likely through increased prepro‐hypocretin mRNA decay. There is some evidence to support that TNF‐α regulates mRNA degradation and stability through the regulation of the AU‐rich element (ARE) binding protein expression.^55^ Our data show that TNF‐α increases p38 phosphorylation, whereas TNF‐α blocking antibody decreases p38 phosphorylation (Figures 4 and 5). It is possible that the effects of TNF‐α on hypocretin/orexin expression are mediated through p38 kinase. It is known that p38 kinase is involved in the regulation of cell phenotypes through modulation of gene expression. p38 regulates gene expression of pro‐inflammatory cytokines^56^ and also regulates interleukin 10 expression through mRNA decay.^57^ Whether TNF‐α regulates the stability of prepro‐hypocretin mRNA through p38 kinase and ARE‐binding proteins remains to be elucidated.

The critical role of hypocretin system in sleep disorders is learned from studies in narcolepsy patients and in knockout mice.^45^, ^58^ Hypocretin system is impaired in narcolepsy patients, and narcolepsy patients have fragmented sleep pattern with excessive daytime sleepiness.^59^, ^60^ Hypocretin (orexin)‐deficient mice show sleep fragmentation with more transitions between wake, non‐rapid eye movement (NREM), and rapid eye movement (REM) sleeps, and a similar phenotype to human narcolepsy including premature entry into REM sleep and poorly consolidated sleep patterns.^43^, ^44^, ^45^ Diseases with excessive daytime sleepiness, including obstructive sleep apnoea, Parkinson's disease, Alzheimer's disease, idiopathic hypersomnia, are associated with increased TNF‐α levels in patient plasma.^19^, ^61^ Mice received repeated TNF‐α treatment showed sleep fragmentation (Figure 6). Our data for the first time demonstrate that TNF‐α directly regulates the homeostasis of hypocretin system by reduction of hypocretin expression. Our data suggest a possible link between elevated TNF‐α level and sleep fragmentation with excessive daytime sleepiness due to impaired hypocretin system.

Our study examined the effects of relatively long‐term or chronic TNF‐α treatment (Figure 6). It is possible that hypocretin system can be influenced in different ways in response to transiently or chronically elevated TNF‐α level, resulting in different sleep‐wake cycle pattern. TNF‐α level is persistently increased in many diseases with excessive day sleepiness with decreased night sleep, such as Parkinson's disease.^18^ Mice receiving repeated TNF‐α treatment for 3 weeks show sleep‐wake cycle fragmentation with decreased NREM sleep (Figure 6). This is intriguing. The traditional thought is that decreased hypocretin (orexin) alone is likely to increase sleep. However, new evidence suggests that the situation can be more complicated. Hypocretin (orexin) knockout mice have fragmented wakefulness and sleep with very short bouts of wake and NREM sleep.^43^ New study suggests that the fragmented behaviour of hypocretin (orexin) knockout mice may due to behavioural state instability with apparently low thresholds to transition between states, instead of a defective arousal system.^43^ We have previously shown that TNF‐α down‐regulates hypocretin receptor 2 (Hcrt2) through a cIAP‐mediated ubiquitination mechanism.^22^ It is possible that HcrtR2 plays a role in this study, in addition to hypocretins, in response to TNF‐α. It is also possible that TNF‐α modulates not only the hypocretin system but also other systems involved in sleep‐wake cycle. Nonetheless, our data suggest that TNF‐α is involved in sleep‐related disorders at least by decreasing hypocretin expression.

Microinjection of hypocretin in the locus coeruleus and pontine inhibitory area results in muscle tone changes,^46^ suggesting that impaired hypocretin system could lead to muscle tone disorder. Muscle activity during REM sleep was induced in mice receiving higher dose of repeated TNF‐α treatment (Figure 6E). Muscle activity (without atonia) during REM sleep is a key feature of REM sleep behaviour disorder (RBD) in patients. Many patients with RBD develop synucleinopathies, such as Parkinson's disease.^62^ The onset of RBD is suggested to precede the development of neurodegeneration by several years.^50^ By retrospective analysis of a small cohort of patients, our data support that RBD exists in Parkinson's disease, Alzheimer's disease and narcolepsy patients, but the percentage of affected patients varies (Figure 6). Our data suggest that TNF‐α involvement in RBD is likely a new insight about the pathophysiological role of TNF‐α. TNF‐α level is increased in Alzheimer's disease patients, and inhibition of TNF‐α is proposed to treat Alzheimer's disease.^63^ Sleep disruption interferes with the normal restorative functions of NREM and REM sleep and is associated with impairments in attention, memory and decision‐making.^64^ Our data indicate that chronically elevated TNF‐α and impaired hypocretin expression are likely involved in the development of impaired learning, memory and cognition. Repeated TNF‐α challenge decreases learning, cognition and memory in mice based on water maze, platform crossings and NOR analysis (Figure 7). We speculate that declined cognition and memory in TNF‐α challenged mice are due to the combined effects of TNF‐α signalling (at least its mediated neuroinflammation) and TNF‐α‐induced reduction of hypocretin. The exact mechanism remains to be elucidated in future studies. So far, little is known about the mechanism by which hypocretin system is regulated during these neurodegenerative disorders.

In conclusion, our work provides evidence that the hypocretin system can be impaired by TNF‐α directly in vivo in mice. The data provide evidence and mechanism that TNF‐α functions as a negative regulator of the hypocretin system in vivo. In addition, repeated TNF‐α challenge induces RBD‐like behaviour and sleep dysfunction, and decreases learning, cognition and memory in mice. This new evidence shed some lights on the pathophysiological role of chronically elevated TNF‐α. Our findings add a new dimension to the pathophysiological role of TNF‐α along with an impaired hypocretin system that may have important implications in health care and neurodegenerative diseases.

## CONFLICT OF INTEREST

The authors confirm that there are no conflicts of interests.

## AUTHORS CONTRIBUTIONS

SZ, PC, XH and QD conceived and designed this study; PC, XKZ, SL, NL, YD, JL, KD, LH and MH performed the research; SZ, PC, XH and QD analysed the data; SZ, NL, YD, JL, ZH, LW, YW, XH and QD contributed materials; and SZ, PC, XH and QD wrote the paper. All authors read and approved the content.

## Data Availability

The data are available from the corresponding author upon reasonable request and subjected to approvals and copyright terms and conditions. Restrictions and approvals may apply to and not limited to the usage, purpose of usage, share and availability of these data.
